# Quantitative analysis of paraspinal muscle atrophy after oblique lateral interbody fusion alone vs. combined with percutaneous pedicle screw fixation in patients with spondylolisthesis

**DOI:** 10.1186/s12891-020-3051-9

**Published:** 2020-01-14

**Authors:** Wei He, Da He, Yuqing Sun, Yonggang Xing, Mingming Liu, Jiankun Wen, Weiheng Wang, Yanhai Xi, Wei Tian, Xiaojian Ye

**Affiliations:** 1 Department of Spine Surgery, Beijing Jishuitan Hospital, Beijing, 100035 China; 2 Department of Spine surgery, Shanghai ChangZheng Hospital, Shanghai, 200003 China

**Keywords:** Oblique lumbar interbody fusion, Percutaneous pedicle screw fixation, Paraspinal muscles atrophy, Functional cross-sectional area, Spondylolisthesis

## Abstract

**Background:**

There is no available literature for comparison on muscle atrophy between the “stand-alone” oblique lateral interbody fusion (OLIF) and regular OLIF (i.e., combined with percutaneous pedicle screws fixation (PPSF) in patients with spondylolisthesis). This study aimed to identify changes in back muscle atrophy between the two surgeries.

**Methods:**

This was a retrospective cohort study of patients who underwent OLIF or OLIF+PPSF at Beijing Jishuitan Hospital and Shanghai ChangZheng Hospital between 07/2014 and 10/2017. Computed tomography (CT) was used to measure functional cross-sectional area (FCSA) and fat infiltration percentage (FIP) of the multifidus and erector spinae before and 24 months after surgery.

**Result:**

There were no differences in FCSA and FIP between OLIF (*n* = 32) and OLIF+PPSF (*n* = 41) groups before surgery. In the OLIF group, the multifidus and erector spinae FCSA and FIP did not change at 24 months (FCSA: multifidus: from 8.59 ± 1.76 to 9.39 ± 1.74 cm^2^, *P* = 0.072; erector spinae: from 13.32 ± 1.59 to 13.55 ± 1.31 cm^2^, *P* = 0.533) (FIP: multifidus: from 15.91 ± 5.30% to 14.38 ± 3.21%, *P* = 0.721; erector spinae: from 11.63 ± 3.05% to 11.22 ± 3.12%, *P* = 0.578). In the OLIF+PPSF group, the multifidus and erector spinae FCSA decreased (multifidus: from 7.72 ± 2.69 to 5.67 ± 1.71 cm^2^, *P* < 0.001; erector spinae: from 12.60 ± 2.04 to 10.15 ± 1.82 cm^2^, P < 0.001), while the FIP increased (multifidus: from 16.13 ± 7.01% to 49.38 ± 20.54%, P < 0.001; erector spinae: from 11.93 ± 3.22% to 22.60 ± 4.99%, *P* < 0.001). The differences of FCSA and FIP between the two groups at 24 months were significant (all P < 0.001). The patients in the standalone OLIF group had better VAS back pain, and JOA scores than the patients in the OLIF combined group (all *P* < 0.05) at 1 week and 3 months after surgery. There were two cases (4.9%) of adjacent segment degeneration in the OLIF combined group, while there was no case in the OLIF alone group.

**Conclusions:**

Standalone OLIF had better clinical outcomes at 1 week and 3 months than OLIF+PPSF in patients with spondylolisthesis. OLIF may not result in paraspinal muscle atrophy at 24 months after surgery.

## Background

Spondylolisthesis is the displacement (usually anterior) of a vertebral body relative to the adjacent inferior vertebral body [1, 2]. It typically affects children but is more symptomatic in adults [3]. Degenerative spondylolisthesis is the most common type in adults [1, 2]. High-grade spondylolisthesis is more common in women [4]. The reported prevalence is 2–6% [2]. Spondylolisthesis is caused by the malfunction of the locking mechanism of the vertebral process between adjacent vertebrae [1]. The most common symptom is back pain; diagnosis can be confirmed by X-rays, magnetic resonance imaging (MRI), and computed tomography (CT) [2, 4]. Prognosis is generally good since 80–90% of the patients report good to excellent outcomes with conservative treatment, but surgery might be necessary for some patients [2, 4].

Interbody fusion is the cornerstone in the surgical treatment of an unstable degenerative lumbar spinal disease, and various techniques have been developed [5, 6]. In recent years, posterior lumbar interbody fusion (PLIF) and transforaminal lumbar interbody fusion (TLIF) have become widely accepted treatments for patients with degenerative spondylolisthesis. Both techniques require extensive dissection of the paraspinal muscles as well as prolonged soft tissue retraction [7]. Complications include significant perioperative bleeding, dural tear, and postoperative muscular atrophy caused by denervation during surgery [8, 9].

The high incidence of paravertebral lumbar muscle injury after open techniques have raised the attention of surgeons for less morbid approaches. Oblique lateral interbody fusion (OLIF) was first introduced in 2012 [10]. Its primary surgical goal is to preserve the posterior column structure, thereby reducing paraspinal muscle trauma. Kim et al. [11] reported that a minimally invasive approach could reduce the markers of muscular injury and systemic inflammatory response. OLIF can be performed as a standalone procedure or in combination with screw fixation [12–14]. Nevertheless, to date, the benefits of standalone OLIF on the paraspinal muscles have not been defined. There is no available literature on the quantitative analysis of the possible difference in back muscle injury and atrophy between standalone OLIF and OLIF combined surgery in patients with spondylolisthesis.

Considering the emerging interest for these techniques, we conducted a retrospective study on the degree of paravertebral lumbar muscle atrophy of standalone OLIF vs. OLIF combined with percutaneous pedicle screws fixation (PPSF) in patients with degenerative spondylolisthesis of grade I operated at one of two hospitals. We sought to identify changes in back muscle atrophy between the two groups, with analysis of the impact of these changes on clinical outcomes.

## Material and methods

### Patient population

This was a retrospective cohort study of patients who underwent standalone OLIF or OLIF combined with PPSF at the Beijing Jishuitan Hospital and Shanghai ChangZheng Hospital between July2014 and October 2017. The study was approved by the ethical committee of the Beijing Jishuitan Hospital (approval number: 201811–03) and the ethical committee of Shanghai Changzheng Hospital (approval number: 201812–01). Informed consent was waived because of the retrospective nature of the study.

The inclusion criteria were:1) underwent standalone OLIF or OLIF combined with PPSF at the L4–5 or L5-S1level for grade I spondylolisthesis [15] with symptoms of radicular pain, intermittent neurogenic claudication, and mechanical low back pain; 2) failure to > 6 months of conservative treatment;3) no previous history of lumbar surgical intervention at the L4–5or L5-S1 level; and 4) available24months of follow-up. The exclusion criteria were: 1) spinal canal stenosis; 2) caudaequina syndrome; 3) spinal tumor; 4) infection in the paraspinal area;5) vertebral fractures; or 6) previous surgery at the L4–5 or L5-S1 level.

### Surgical procedures

All patients were operated by surgeons who had > 20 years of experience in spinal surgery. In the standalone OLIF group, OLIF surgery was performed according to the standard procedure [16]. Briefly, under general anesthesia, the patients were placed in the lateral decubitus position on their right side, and the target intervertebral disc space was identified under fluoroscopic guidance. The presence of scoliosis does not affect the side of surgical approach. A 4-cmskin incision was made 6–10 cm anterior to the mid-portion of the marked disc. The surgical team approached the retroperitoneal space via blunt dissection and by mobilizing the peritoneum anteriorly to expose the anatomical oblique lateral corridor, followed by intervertebral cage insertion (Clydesdale spinal system, Medtronic, Memphis, TN, USA; 12 × 50 × 18 mm, 6° lordotic, 3.27 cc graft volume) filled with demineralized bone matrix (DBM) (Wright Medical Technology Inc., Arlington, TN, USA).

In the OLIF combined with the PPSF group, OLIF was performed based on the standard procedure [16]. After fusion, the patients were placed in the prone position to undergo posterior bilateral PPSF (CD Horizon Solera Voyager Spinal System; Medtronic, Memphis, TN, USA).

None of the patients in both groups underwent additional laminectomy at the index level. Operative time and blood loss were recorded.

### Radiological assessment

To assess the degree of paravertebral lumbar muscle atrophy of the patients of the two groups, we used the functional cross-sectional area (FCSA) protocol, as previously described [17, 18]. Lumbar CT was performed using an Aquilion 64-slice scanner (Toshiba, Tokyo, Japan) before and 24 months after surgery. Using 5-mm thick slices, images were obtained with patients placed in the spine position. Images were stored in a digital imaging and communications in medicine (DICOM) format and analyzed on a personal computer using the Tissue Composition Module of the software (Mindways, Austin, TX, USA). The multifidus and erector spinae were measured in selected axial images at the lower third of the vertebral body(L3) above the operation level(L4/5) to avoid the artifact produced by the screws. Avoiding nearby fat, bony structures, and other soft tissues, the region of interest (ROI) was drawn with an electronic pencil. FCSA and fat infiltration percentage (FIP) of multifidus and erector spinae on the right side before surgery and 24 months after surgery were measured automatically by the software (Additional file 1: Figure S1). Adjacent segment degeneration was defined as narrowing intervertebral space and the disagreement of bilateral intervertebral space.

### Data collection

History of osteoporosis (defined as T-score < − 2.5) was verified from the medical charts. The clinical outcomes were based on the visual analog scale (VAS) score for pain and the Japanese Orthopaedic Association (JOA) score at 1 week, 3 months, and 2 years after operation. Erector spinae and multifidus were examined before surgery and 2 years after surgery. Degeneration of the adjacent segment was examined at 2 years after surgery.

### Statistical analysis

Data analysis was performed using SPSS 18.0 (IBM, Armonk, NY, USA). Continuous variables are expressed as means ±standard deviation and were analyzed using the Student’s t-test. VAS and JOA scores were analyzed using repeated measure ANOVA with the post hoc paired samplet-test. Categorical variables are expressed as number (percentage) and were analyzed using Pearson’s chi-square test or Fisher’s exact test, as appropriate. A *P* value < 0.05 was considered statistically significant.

## Results

### Characteristics of the patients and operation data

Finally, 73 patients were included. The characteristics of the patients are presented in Table 1. The standalone OLIF group included 10 men (31.2%) and 22 women (68.8%), with a mean age of 59.8 ± 13.7 years (range, 38 to 88). Among the 32 cages inserted, 30 (93.8%) were located at the L4–5 level, and two (6.2%) at L5-S1 level. The OLIF combined group included 11 men (26.8%) and 30 women (73.2%), with a mean age of 61.0 ± 9.3 years (range, 44 to 86). Among the 41 cages inserted, 37 (90.2%) were located at the L4–5 level and four (9.8%) at L5-S1 level. And no patient received arthrodesis in both groups. The operative time was significantly shorter in the standalone OLIF group than in the OLIF combined group (98 ± 14 vs. 182 ± 32 min, *P* < 0.001). There was significantly less blood loss in the standalone OLIF group than in the OLIF combined group (108 ± 49 ml vs. 140 ± 36 ml, *P* = 0.002).

**Table 1:** Characteristics of the patients

	OLIF alone (*n* = 32)	OLIF+PPSF (*n* = 41)	P
Gender			
Male	10 (31.3%)	11 (26.8%)	0.679
Female	22 (68.7%)	30 (73.2%)	
Age (years)	59.8 ± 13.7	61.0 ± 9.3	0.669
Level			
L4–5	30 (93.8%)	37 (90.2%)	0.584
L5-S1	2 (6.2%)	4 (9.8%)	
Osteoporosis	8 (25.0%)	15 (36.6%)	0.290
Operative time (min)	98 ± 14	182 ± 32	< 0.001
Blood loss (mL)	108 ± 49	140 ± 36	0.002

*OLIF* Oblique lateral interbody fusion, *PPSF* Percutaneous pedicle screw fixation

### Clinical outcomes

**Fig. 1:**
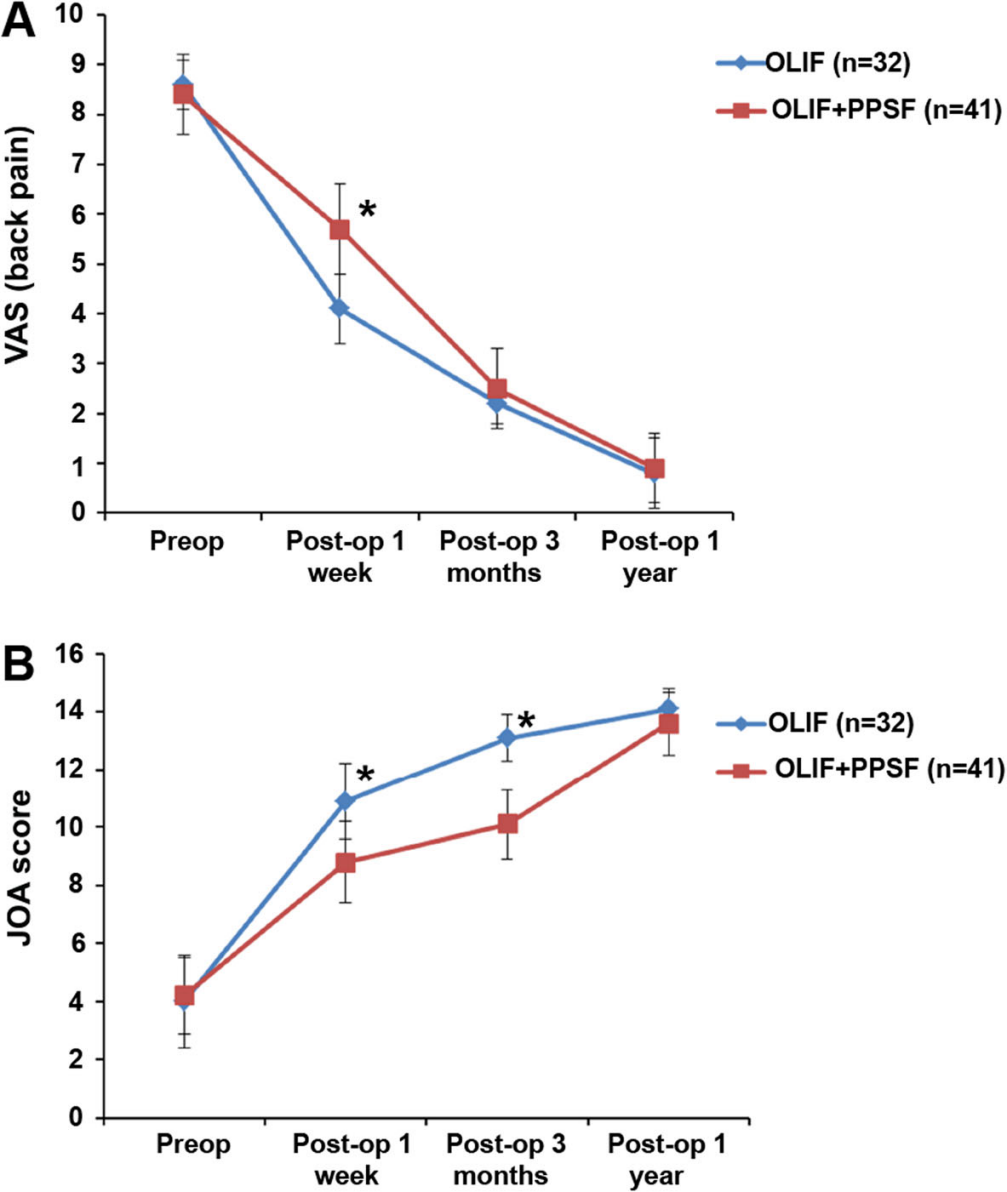
Visual analog score (VAS) (**a**) of back pain and the Japanese Orthopaedic Association (JOA) score (**b**) before and after operation. OLIF: oblique lateral interbody fusion; PPSF: percutaneous pedicle screws fixation. *There were significant differences between the two groups at one week and three months after surgery

**Fig. 2:**
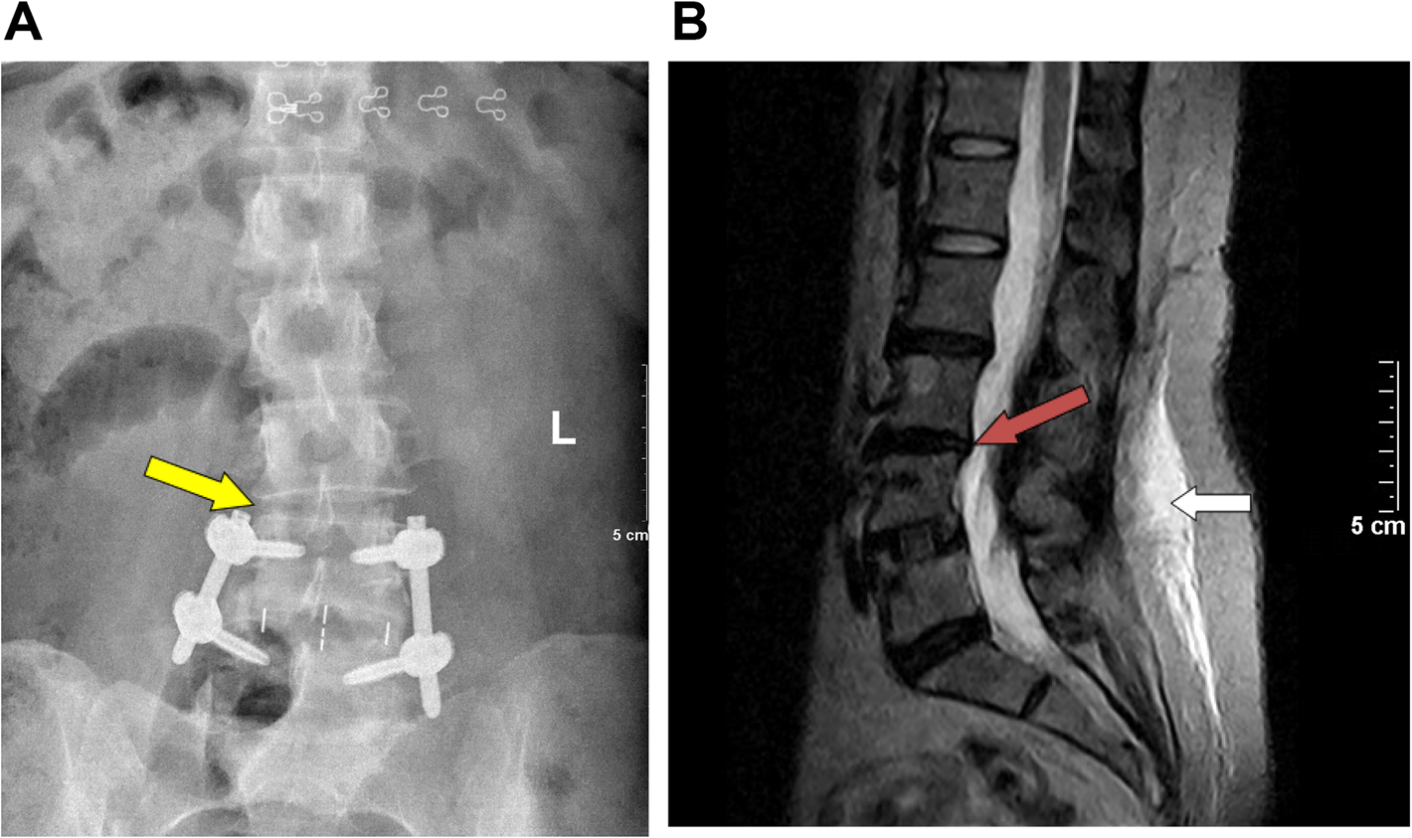
**a** The disagreement regarding the bilateral intervertebral space. One side of the intervertebral height space at L3/4 is significantly smaller than on the other side (yellow arrow). **b** The red arrow indicates that the trailing edge of the L3 vertebral body above the L3/4 disc protrudes backward from the posterior edge of the L4 vertebral body, suggesting that adjacent segment degeneration has occurred. The white arrow indicates postoperative edema of the fascia behind the lumbar spine in the regular OLIF group

The two groups showed significant improvement in all clinical outcome scores at all time points after surgery compared to preoperative data. The patients in the standalone OLIF group had better VAS back pain, and JOA scores than the patients in the OLIF combined group (all *P* < 0.05) at 1 week and 3 months after surgery (Fig. 1, Table 2). There were two cases (4.9%) (Fig. 2) of adjacent segment degeneration in the OLIF combined group, while there was no case in the OLIF alone group. There was no significant difference in adjacent segment degeneration in the standalone OLIF group after surgery. In addition, no patient occurred implant migration in either group.

**Table 2:** Surgical outcomes

	OLIF (*n* = 32)	OLIF+PPSF (*n* = 41)	*P*
VAS (back pain)			
Pre-op	8.6 ± 0.5	8.4 ± 0.8	0.241
Post-op 1 week	4.1 ± 0.7	5.7 ± 0.9	< 0.001
Post-op 3 months	2.2 ± 0.4	2.5 ± 0.8	0.030
Post-op 2 years	0.8 ± 0.7	0.9 ± 0.7	0.831
JOA score			
Pre-op	4.0 ± 1.6	4.2 ± 1.3	0.636
Post-op 1 week	10.9 ± 1.3	8.8 ± 1.4	< 0.001
Post-op 3 months	13.1 ± 0.8	10.1 ± 1.2	< 0.001
Post-op 2 years	14.1 ± 0.7	13.6 ± 1.1	0.055
Adjacent segment degeneration	0	2 (4.9%)	0.125

*OLIF* Oblique lateral interbody fusion, *PPSF* Percutaneous pedicle screw fixation, *VAS* Visual analog scale, *JOA* Japanese Orthopaedic Association

### Functional cross-sectional area of the multifidus and erector spinae

**Fig. 3:**
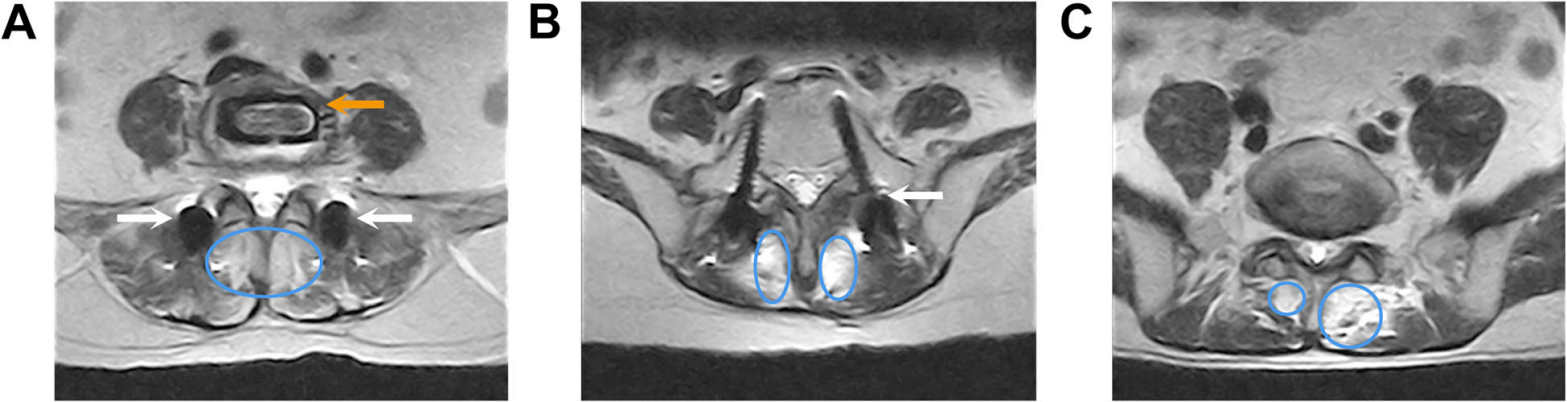
Iatrogenic paravertebral muscle injury at fusion cage level (**a**); pedicle screw level (**b**) and adjacent segment level (**c**). A large number of non-muscle tissues (high signal area in the blue circle) occupying the original muscle position 2 years after surgery. The white arrow indicates the pedicle screw. The orange arrow indicates the intervertebral cage

The mean FCSA measurements are presented in Table 3. There were no differences between the two groups before surgery. In the standalone OLIF group, the meanmultifidus and erector spinae FCSA did not change at 24 months (multifidus: from 8.59 ± 1.76 to 9.39 ± 1.74 cm^2^, *P* = 0.072; erector spinae: from 13.32 ± 1.59 to 13.55 ± 1.31 cm^2^, *P* = 0.533). In the OLIF combined group, the mean multifidus and erector spinae FCSA decreased after surgery (multifidus: from 7.72 ± 2.69 to 5.67 ± 1.71 cm^2^, *P* < 0.001; erector spinae: from 12.60 ± 2.04 to 10.15 ± 1.82 cm^2^, *P* < 0.001). The differences between the two groups at 24 months were significant (all *P* < 0.001). Figure 3 shows an iatrogenic muscle injury.

**Table 3:** Functional cross-sectional area of the multifidus and erector spinae before and 24 months after surgery

	OLIF (*n* = 32)	OLIF+PPSF (*n* = 41)	*P* ^a^
Multifidus (cm^2^)			
Pre-op	8.59 ± 1.76	7.72 ± 2.69	0.099
Post-op 2 years	9.39 ± 1.74	5.67 ± 1.71	< 0.001
P^b^	0.072	< 0.001	
Erector spinae (cm^2^)			
Pre-op	13.32 ± 1.59	12.60 ± 2.04	0.104
Post-op 2 years	13.55 ± 1.31	10.15 ± 1.82	< 0.001
P^b^	0.533	< 0.001	

*OLIF* Oblique lateral interbody fusion, *PPSF* Percutaneous pedicle screw fixation

^a^ Comparison between the two groups

^b^ Comparison between pre- and post-operation

### Fat infiltration percentage of the multifidus and erector spinae

The mean FIP measurements are presented in Table 4. There were no differences between the two groups before surgery. In the standalone OLIF group, the mean multifidus and erector spinae FIP did not change at 24 months (multifidus: from 15.91 ± 5.30% to 14.38 ± 3.21%, *P* = 0.721; erector spinae: from 11.63 ± 3.05% to 11.22 ± 3.12%, *P* = 0.578). In the OLIF combined group, the mean multifidus and erector spinae FIP increased after surgery (multifidus: from 16.13 ± 7.01% to 49.38 ± 20.54%, *P* < 0.001; erector spinae: from 11.93 ± 3.22% to 22.60 ± 4.99%, *P* < 0.001). There were significant differences between the two groups at 24 months (all *P* < 0.001).

**Table 4:** Fat infiltration percentage of the multifidus and erector spinae before and 24 months after surgery

	OLIF (*n* = 32)	OLIF+PPSF (*n* = 41)	P ^a^
Multifidus (%)			
Pre-op	15.91 ± 5.30	16.13 ± 7.01	0.885
Post-op 2 years	14.38 ± 3.21	49.38 ± 20.54	< 0.001
P ^b^	0.721	< 0.001	
Erector spinae (%)			
Pre-op	11.63 ± 3.05	11.93 ± 3.22	0.686
Post-op 2 years	11.22 ± 3.12	22.60 ± 4.99	< 0.001
P ^b^	0.578	< 0.001	

*OLIF* Oblique lateral interbody fusion, *PPSF* Percutaneous pedicle screw fixation

^a^ Comparison between two groups

^b^ Comparison between pre- and post-operation

## Discussion

Although conventional open TLIF or PLIF plus pedicle screw fixation are beneficial for the surgical treatment of spondylolisthesis, many factors might cause iatrogenic and degenerative changes to the back muscle after spinal fusion [19, 20]. OLIF addresses these issues because of the anterior approach [10], but PPSF can still be associated with muscle injury. Percutaneous pedicle screws are fixed to the vertebral pedicle throughout the subcutaneous fascia, paraspinal muscles, and articular processes. Moreover, the pedicle screw tail occupies a certain muscle volume. At the same time, the two screws (upper and lower vertebral bodies) need to be connected by a titanium rod, and the titanium rod is placed in the muscle layer. All the operations to place these devices cause damage to the paravertebral muscles and cause muscle edema. Muscle damage can cause pain in the surgical site. There is no available literature on back muscle injury and atrophy between standalone OLIF and OLIF combined with PPSF in patients with spondylolisthesis. Therefore, this study aimed to identify changes in back muscle atrophy between the two groups. The results suggest that standalone OLIF may result in less important paraspinal muscle atrophy than OLIF combined with PPSF in patients with spondylolisthesis. This could contribute to better clinical outcomes at 24 months after surgery. Nevertheless, the faster recovery observed in the standalone OLIF group at least suggests that the patients can recover a normal life earlier than those with OLIF combined with PPSF.

One of the most important functions of the trunk muscles is to support the vertebral body. In particular, the extensor muscle of the lower lumber part plays a vital role in maintaining the balance of the lumbar levels and is also a dynamic stabilizer for the movement of the spine-pelvis complex. Atrophy of the back muscle correlates with clinical outcomes after vertebral fusion [21, 22]. Therefore, assessing muscle atrophy after spine surgery could help predict the outcomes. Hu et al. [23] demonstrated that FCSA assessed by CT is an acceptable method for evaluating paraspinal muscle atrophy, with a high consistency of 0.794 in the intraclass correlation between CT and MRI. Many studies have reported a decrease in the adjacent FCSA, ranging from − 2 to − 38% using an open approach, compared with + 9.9% to − 12.2% using minimally invasive techniques [17, 24–26].

Kawaguchi et al. [27] reported that back muscle injury after posterior lumbar surgery is related to the operation time and retraction pressure. Therefore, he recommended that the retraction be released for 5 min after 1 h of retraction to prevent serious back muscle injury. Gejo et al. [28] also reported that the muscle retraction time influences the postoperative back muscle function. Datta et al. [29] and Taylor et al. [30] reported that > 2 h of retraction reduced the flow of capillary vessels, causing ischemic intramuscular changes.

Hyun et al. [31] reported that the paramedian interfascial approach might preserve the back muscle according to the midline and paramedian approaches in lumbar fusion, according to CT data. Therefore, OLIF has attracted considerable attention because OLIF uses the ante-psoas muscle approach, which theoretically avoids paraspinal muscle damage and decreases the risk of chronic low back pain. Nevertheless, CT is not appropriate to estimate the muscles at the fusion level on account of the interference by the metal artifacts [31, 32]. Therefore, methods to quantify muscle atrophy have been examined with the aim of analyzing the degree of paraspinal muscle atrophy [18]. In this study, standalone OLIF achieved better clinical outcomes (VAS and JOA scores) over the first 24 months after surgery compared with OLIF combined with PPSF.

These better clinical outcomes were probably associated with the better FCSA, and FIP found in the standalone OLIF group compared with the OLIF combined group. This observation is probably due to no invasion of the paraspinal muscles with standalone OLIF. Fan et al. [7] reported that there were significant differences not only in back pain VAS but also in the Oswestry disability index when they used the paramedian interfascial approach compared to the midline approach. Kim et al. [32] demonstrated that the atrophy of multifidus muscles appeared less important and trunk extension muscle strength was better preserved in patients who underwent PPSF compared to patients who received open surgery. Paraspinal muscles surrounding the surgical site are damaged due to iatrogenic denervation, as observed for the MF [33] and longissimus [34, 35] muscles, which are innervated by the medial branch nerve and in part by the intermediate branch nerve of the posterior rami. In a cadaver study, a medial branch nerve affection rate of84% was described when inserting a screw via the mini-open surgical approach [36]. Accordingly, Hu et al. [37] also reported atrophy of the back muscle due to denervation of the paraspinal muscle, based on the results of a small animal study. Because the medial and intermediate branch nerves have the same origin, which is lateral to the facet joints and superior to the transverse processes [34], the insertion of pedicle screws may damage both nerves.

There were two cases of adjacent segment degeneration in the OLIF combined group, but none in the standalone OLIF alone. We believe that strong fixation results in a long-term disuse paraspinal muscle atrophy and further accelerate the degeneration of adjacent segments. First, the process of placing the pedicle screw and the titanium rod affects the small facet joints of the adjacent segments. Second, paravertebral muscles are damaged, and muscle strength is reduced, which may accelerate the instability of adjacent segments. Strube et al. [26] reported that the overall atrophy and fatty degeneration ratio at the adjacent segments after the anterior-only treatment option (ALIF) appears to be rather low. Regarding motion-preserving surgical treatment such as total disc replacement, a significantly lower ratio of adjacent-segment degeneration was reported compared with the fusion approach [38]. Interestingly, a higher rate of adjacent-segment degeneration was also observed for a transpedicular stabilized fixation where a posterior surgical approach was used [39]. Nevertheless, unilateral fixation has a lower incidence of degeneration than bilateral fixation in the proximal stage. BPS fixation was found to be overrigid, which would cause device-related osteoporosis, absorption of grafted bone, and degeneration of adjacent segments [39–41]. According to previous studies and the results of the present study, regarding the development or progression of adjacent-segment degeneration, we think that it may be predominantly related to muscle atrophy as well as over-rigid fixation.

The present study suggests that even though OLIF combined with PPSF can be done using a mini-open or percutaneous technique, there is still a risk of paraspinal muscle denervation. These findings agree with previous studies. Regev et al. [36] reported that percutaneous screw application could potentially reduce the indirect damage caused to the medial branch nerve from 84% to 20%. Alternative surgical approaches such as stand-alone ALIF [42] or different screw entry points such as cortical screw-rod fixation [43] would completely avoid affection of the medial and intermediate branch nerves. Other studies showed that iatrogenic alterations decrease postoperative cross-sectional area (CSA) and contractile tissue density of back muscles [20, 44, 45].

In this study, FIP of multifidus and erector spinae at 2 years after surgery was higher than before surgery in the OLIF combined group. The reason could be that the multi-cleft muscle is adjacent to the vertebral spinous process. Due to the compression of the percutaneous pedicle screw channel to the midline during the operation, the multifidus is sandwiched between the spinous process and the channel, so the internal part of the fissure muscle is more prone to edema. On the other hand, there was no difference in FIP of the two muscles in the standalone OLIF group after surgery. This result is consistent with the FCSA results. Therefore, we believe that an increase in the fat percentage in the paravertebral muscle is another important factor in accelerating muscle atrophy after fusion.

This study has limitations. The number of patients was small. This was a retrospective study with all the inevitable biases. There was no randomization, and a variety of factors could have affected the selection of the surgical approach. Finally, factors such as general condition, comorbidities, and neurological conditions were not considered. Further studies are required to confirm whether muscle injury is directly related to the long-term clinical outcome.

## Conclusion

In conclusion, standalone OLIF can achieve better clinical outcomes compared with OLIF combined with PPSF for grade I spondylolisthesis, as shown by less back pain at 1 week and 3 months after operation. OLIF may not result in paraspinal muscle atrophy at 24 months after surgery.

## Supplementary information

**Additional file 1: Figure S1.** Measurement of the functional cross-sectional area and fat infiltration percentage. Green circles show the multifidus (A) and erector spinae (B). The functional cross-sectional areas and fat infiltration percentageswere measured automatically by the software.

## Data Availability

The datasets used and/or analyzed during the current study are available from the corresponding author on reasonable request.

## Abbreviation

CT: Computed tomography
FCSA: Functional cross-sectional area
FIP: Fat infiltration percentage
MRI: Magnetic resonance imaging
OLIF: Oblique lateral interbody fusion
PLIF: Posterior lumbar interbody fusion
PPSF: Percutaneous pedicle screws fixation
TLIF: Transforaminal lumbar interbody fusion
