# Supplementary figures and images for: Quantitative analysis of paraspinal muscle atrophy after oblique lateral interbody fusion alone vs. combined with percutaneous pedicle screw fixation in patients with spondylolisthesis
Source: BMC Musculoskelet Disord. 2020 Jan 14;21:30. doi: 10.1186/s12891-020-3051-9 (PMC6961348; doi:10.1186/s12891-020-3051-9)

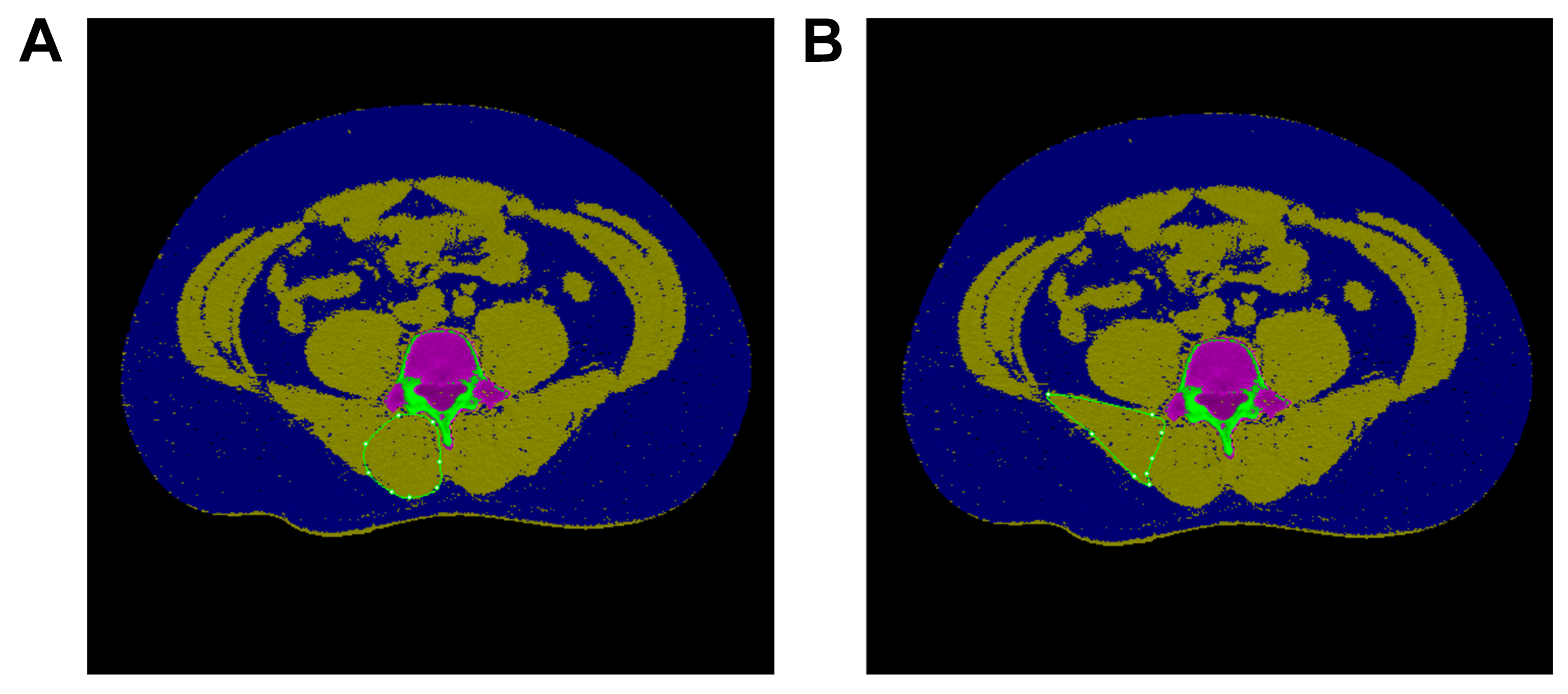

Supplement: Supplementary file 1 — Additional file 1: Figure S1. Measurement of the functional cross-sectional area and fat infiltration percentage. Green circles show the multifidus (A) and erector spinae (B). The functional cross-sectional areas and fat infiltration percentageswere measured automatically by the software. [file 12891_2020_3051_MOESM1_ESM.tif]
